# The route to solve the interplay between inflammation, angiogenesis and anti-cancer immune response

**DOI:** 10.1038/cddis.2016.211

**Published:** 2016-07-21

**Authors:** C Botta, G Misso, E C Martino, L Pirtoli, M G Cusi, P Tassone, P Tagliaferri, M Caraglia, P Correale

**Affiliations:** 1 Department of Clinical and Experimental Medicine, Magna Graecia University, Catanzaro, Italy; 2 Department of Biochemistry, Biophysics and General Pathology, Second Naples University, Naples, Italy; 3 Department of Oncology, Siena, Italy; 4 Department of Medical Biotechnology University of Siena, Siena, Italy

Even though the crucial role played by inflammation in cancer development and progression was first hypothesized by Rudolf Virchow at the beginning of the nineteenth century, only recently inflammation has been recognized as a hallmark of cancer. At present, the biology underlying the humoral and cellular immune-suppressive cancer-associated inflammatory microenvironment is an active area of preclinical and clinical investigation.^1, 2^ Indeed, the possibility to modulate the inflammatory/immune microenvironment, by either antagonizing the tumor-associated immune-suppression or by enhancing the pre-existing anti-cancer immune response in tumor tissues, is a promising therapeutic option for cancer patients. In this context, the presence of infiltrating lymphocytes with specific immune-phenotypes within the tumor or in the surrounding stroma has predicted long-lasting responses and improved patients' survival. Indeed, in a series of homogenously treated metastatic colorectal cancer (mCC) patients, we observed longest survival when high CCR7^+^ or FoxP3^+^ (T_reg_) lymphocytes infiltration occurred within tumor tissues. This latter finding provided a 'paradigm shift', since for the first time associated the density of an immune-suppressive population with a better outcome in mCC patients. This finding may be now explained by the recently disclosed 'regulatory' rather than 'suppressive' nature of T_reg_s. In this vision, in fact, these cells may work as specific repressor of the IL-17/Th_17_-driven inflammation, produced by alteration in the gut 'microbiota', and this event, in turn, may account for the survival benefit.^3, 4, 5^ Accordingly, we reported that a high baseline inflammatory status negatively affects patients' prognosis and impairs the response to standard and immune-modulatory anti-cancer treatments in different malignancies including colorectal cancer, multiple myeloma and non-small cell lung cancer (NSCLC).^6, 7, 8, 9^ In our latest report, recently published in *Cell Death Discovery,*^10^ we added a new piece to the puzzle (Figure 1), by investigating the immune-modulatory properties of bevacizumab, a monoclonal antibody (mAb) that acts by sequestering VEGFA, in NSCLC patients enrolled in mPEBev (BEVA2007) trial; our findings provide novel evidence of a functional link between angiogenesis and the immune/inflammatory response.^6, 11, 12^ Our work has been designed taking into account that the biological relevance of VEGFA is not limited to endothelial cells, and consequently to angiogenesis. Indeed, this molecule, produced and released by tumor cells, platelets and inflammatory cells, such as neutrophils and monocytes, in the cancer-associated inflammatory microenvironment, attracts myeloid-derived suppressor cells and tumor-associated macrophages, and impairs the immune-system by counteracting the activity of dendritic cells (DCs) and specific T-cell subsets.^13^ In the BEVA2007 clinical study, we evaluated the efficacy and safety of the mPE (cisplatinum+metronomic oral etoposide)+bevacizumab schedule as frontline therapy in a series of NSCLC patients. We found that this regimen is safe and highly active. Furthermore, according to the immune-modulatory role of VEGFA, we observed that patients who presented a low inflammatory status at baseline, as indicated by a low neutrophil-to-lymphocyte ratio (NLR), experienced a 20 months median overall survival with an impressive 29% hazard ratio.^6^ In our recently published paper, we added novel information demonstrating that the addition of bevacizumab to chemotherapy in NSCLC patients decreases systemic inflammatory status and promotes anticancerimmune-modulating effects. Specifically, we observed a significant reduction in the levels of proangiogenic factors (VEGFA, Angiopoietin 2 and Follistatin) and inflammatory cytokines (IFNγ, IL4 and IL17A) in the serum of these patients and a progressive decline in NLR. Additionally, we found that both mPE and mPE+bevacizumab (mPEBev) regimens induced a significant increase of central memory T lymphocytes in peripheral blood, while only patients who underwent mPEBev treatment presented a significant rise in the percentage of mature DCs. The latter finding is suggestive of improved antigen presentation consequent to bevacizumab-dependent VEGFA deprivation. These data were also in line with a functional *ex vivo* study performed on antigen-specific cytotoxic T lymphocyte (CTL) lines generated *in vitro* from peripheral blood mononuclear cells at baseline and after mPE or mPEBev treatment. In particular, antigen-specific CTL proliferation and Th1 polarization was more pronounced in T-cell lines derived from patients who received the mPEBev regimen as compared with T cells lines derived from patients who received chemotherapy alone. On these bases, we hypothesized that the anti-tumor activity of bevacizumab may at least in part rely on the modulation of the immune/inflammatory response.

The next step to decipher the interplay between inflammation, angiogenesis and immune response relies on the understanding of the events that occur at the deep molecular level. Genomic instability, epigenetic and noncoding RNA networks are currently under active investigation to shed light on the fine tuning of the host immune/inflammatory response against cancer and the balance between immune activation and immune-suppression, in order to identify new potential therapeutic targets to improve cancer patients outcome.

## Figures and Tables

**Figure 1:**
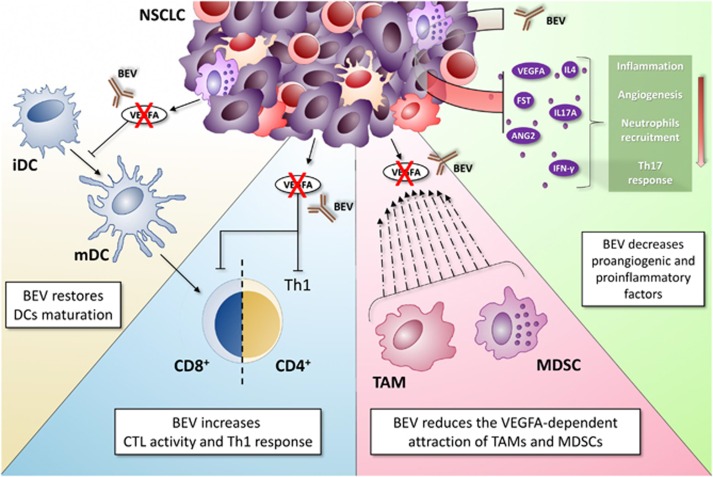
Summary of the main bevacizumab-mediated immunomodulatory events. VEGF-A inhibition by bevacizumab has been correlated with a number of immunological events. Bevacizumab is able to promote DCs maturation, as well as to improve DCs' amount and function. In addition, this monoclonal antibody can improve both CD8^+^ CTLs and CD4^+^ T helper 1 cells (Th1) immune response in tumor surveillance. In addition, as part of the immunomodulatory events induced by VEGFA neutralization, bevacizumab leads to a significant inhibition of MDSCs and TAM accumulation in tumor microenvironment. Finally, the expression levels of several proangiogenic and proinflammatory factors appear strongly reduced upon bevacizumab treatment. CTLs, cytotoxic T cells; DCs, dendritic cells; MDSCs, myeloid-derived suppressor cells; TAM, tumor-associated macrophages; VEGF-A, vascular endothelial growth factor A
